# A brief intervention for preparing ICU families to be proxies: A phase I study

**DOI:** 10.1371/journal.pone.0185483

**Published:** 2017-10-02

**Authors:** Alison E. Turnbull, Caroline M. Chessare, Rachel K. Coffin, Dale M. Needham

**Affiliations:** 1 Outcomes After Critical Illness and Surgery (OACIS) Group, Johns Hopkins University, Baltimore, Maryland, United States of America; 2 Division of Pulmonary and Critical Care Medicine, School of Medicine, Johns Hopkins University, Baltimore, Maryland, United States of America; 3 Department of Epidemiology, Bloomberg School of Public Health, Johns Hopkins University, Baltimore, Maryland, United States of America; 4 Medical Intensive Care Unit, Johns Hopkins Hospital, Baltimore, Maryland, United States of America; 5 Department of Physical Medicine and Rehabilitation, School of Medicine, Johns Hopkins University, Baltimore, Maryland, United States of America; University of Pittsburgh, UNITED STATES

## Abstract

**Background:**

Family members of critically ill patients report high levels of conflict with clinicians, have poor understanding of prognosis, struggle to make decisions, and experience substantial symptoms of anxiety, depression, and post-traumatic stress regardless of patient survival status. Efficient interventions are needed to prepare these families to act as patient proxies.

**Objectives:**

To assess a brief “patient activation” intervention designed to set expectations and prepare families of adult intensive care unit (ICU) patients to communicate effectively with the clinical team.

**Design:**

Phase I study of acceptability and immediate side effects.

**Setting and participants:**

122 healthcare proxies of 111 consecutive patients with a stay of ≥24 hours in the Johns Hopkins Hospital Medical ICU (MICU), in Baltimore, Maryland.

**Intervention:**

Reading aloud to proxies from a booklet (Flesch-Kincard reading grade level 3.8) designed with multidisciplinary input including from former MICU proxies.

**Results:**

Enrolled proxies had a median age of 51 years old with 83 (68%) female, and 55 (45%) African-American. MICU mortality was 18%, and 37 patients (33%) died in hospital or were discharged to hospice. Among proxies 98% (95% CI: 94% - 100%) agreed or strongly agreed that the intervention was appropriate, 98% (95% CI: 92% - 99%) agreed or strongly agreed that it is important for families to know the information in the booklet, and 54 (44%, 95% CI 35%– 54%) agreed or strongly agreed that parts of the booklet are upsetting. Upset vs. non-upset proxies were not statistically or substantially different in terms of age, sex, education level, race, relation to the patient, or perceived decision-making authority.

**Conclusions:**

This patient activation intervention was acceptable and important to nearly all proxies. Frequently, the intervention was simultaneously rated as both acceptable/important and upsetting. Proxies who rated the intervention as upsetting were not identifiable based on readily available proxy or patient characteristics.

## Introduction

The proportion of Medicare beneficiaries mechanically ventilated during the last month of life has risen steadily over the last 10 years.[1,2] The majority of these patients lack decision-making capacity and reply on family members to communicate with the intensive care unit (ICU) clinical team.[3,4] These family members report high levels of conflict with clinicians,[5,6] have poor understanding of prognosis,[7–10] struggle to make decisions,[11–13] and experience substantial symptoms of anxiety, depression[14,15], and post-traumatic stress [16–19] regardless of patient survival status. In response to these challenges, the Society of Critical Care Medicine and the American Thoracic Society have endorsed clinical practice guidelines on family-centered care in the ICU[20] and engaging family members in shared decision making[21].

ICU clinicians commonly report that family members’ incorrectly optimistic expectations for patient recovery is a major challenge to effective communication [22–25]. Effective communication with families is also time-consuming, not consistently taught during critical care training programs[26], and requires advanced communication skills [27,28]. It is therefore unsurprising that prompt, multi-disciplinary family meetings have not been widely adopted[29,30] despite their endorsement as an indicator of high-quality ICU care [31].

Against this background, we recognize that family members of incapacitated patients have strong incentives to ensure that communication with ICU clinicians is clear and meaningful. “Patient activation” is a type of intervention that seeks to equip patients with the skills and confidence required to effectively engage healthcare providers for purposes of clinical decision-making [32,33]. Although traditionally used directly with patients in the outpatient setting, patient activation can be adapted to provide a similar intervention for ICU families. Therefore, we designed an intervention to help set appropriate expectations about patient recovery, explain the role of a healthcare proxy in the ICU, and prepare families to communicate effectively with the ICU clinical team. We recognize that family members prefer different decision-making roles and that some family members have strong emotional or psychological aversions to assuming decision-making responsibility[34]. Hence, this evaluation was designed as a Phase I study to assess the acceptability and immediate side effects of this brief activation intervention for ICU families.

## Methods

### Intervention design

The intervention was designed to 1) help set expectations about patient recovery from critical illness 2) educate adult family members in the medical intensive care unit (MICU) at Johns Hopkins Hospital (JHH) about the role of a healthcare proxy and 3) prepare proxies to communicate effectively with their ICU clinical team. Input from multi-disciplinary stakeholders was solicited during design of the intervention. First, as part of a qualitative study of physician-perceived facilitators and barriers to discussing post-hospitalization outcomes with ICU families,[24] intensivists at 20 hospitals across the U.S. were asked to describe the behaviors of effective ICU proxies, and to suggest questions that proxies should ask intensivists. We used these responses to draft an initial version of a short booklet and elicited feedback from the following JHH stakeholders: the JHH Patient and Family Advisory Council, MICU social workers, risk management (legal counsel), patient relations, chaplaincy and spiritual care, the MICU end-of-life interest group (primarily comprised of nurses), MICU nurse practitioners and physician assistants, as well as pulmonary and critical care fellows and MICU attending physicians. Iterative changes were made based on feedback throughout this process. The families of 6 current MICU patients who were not enrolled in the study also were asked to provide feedback.

The resulting intervention consisted of reading aloud from the final version of the booklet and then providing participants with the booklet for review. The 554-word booklet used in this study required 3.5 minutes to read aloud, with a Flesch-Kincard reading grade level of 3.8 and Flesch reading ease score of 85. The booklet contains black-and-white icons designed to be recognizable to a socio-economically and racially diverse population [35], and is freely available for download under at www.piperscience.org/proxy-activation. All interactions with study participants, including recruiting, consenting, and delivering the intervention were performed by white females (A.E.T., C.M.C., R.K.C.) of similar age (25–35 years old).

### Study design

This investigation was designed as a Phase I study to assess the intervention’s acceptability to ICU families and identify immediate side effects [36]. We assessed acceptability by asking all participants to respond to the following two statements using a 5-point Likert scale ranging from Strongly agree to Strongly disagree: 1) “*The booklet is* ***appropriate*** *for adult friends and family-members of ICU patients*.*”* 2) “*It is* ***important*** *for families of ICU patients to know the information in the booklet*.*”* On an *a priori* basis, we defined the intervention as acceptable if ≥80% of participants described the intervention as both appropriate and important for adult friends and family members of ICU patients. Study sample size was designed to estimate the proportions of ICU proxies who rated the intervention as appropriate and important with a 5% margin of error (95% confidence interval width of 10%). We also anticipated that participants might find information in the booklet upsetting at the time of the intervention and treated this as a potential immediate side-effect. This outcome was evaluated by asking all participants to respond to the statement *“Parts of the booklet are* ***upsetting****”* using the same 5-point Likert scale.

### Recruitment

All recruitment took place in the JHH MICU, located in the inner city of Baltimore, Maryland between January and May 2016. The MICU census was screened 7 days per week to identify all consecutive eligible study participants. Family members[20] became eligible for recruitment as soon as their loved one had been a patient in the MICU for 24 hours. When multiple members of the family were available to participate the study team members enrolled the patient’s health agent or surrogate. Evaluation of this recruitment strategy have been previously published.[37] In this paper we refer to these people as proxies to encompass both healthcare agents and healthcare surrogates who have distinct legal definitions under Maryland state law.[38,39] If the family member initially enrolled was not the patient’s legal healthcare proxy, additional attempts were made to also enroll a legal proxy. Interviews were conducted in private spaces within the MICU but outside the patient’s room. Participants provided oral, in-person consent, which was documented using a standardized consent form and received a $10 gift card upon interview completion. Additional information about family recruitment and data collection are provided in Table A of S1 File. Johns Hopkins Medicine IRB Committee X approved this study number: IRB00080137. The IRB deemed oral consent as appropriate because participating in the study did not involve any procedure for which written consent is normally required outside of the research context.

### Analyses

The primary outcomes were the proportions of participants who agreed or strongly agreed that the intervention is appropriate and that it is important for families of ICU patients to know the information conveyed by the intervention. Starting during the third week of the study, participants who agreed or strongly agreed that the information was important were asked to identify the most important pages in the booklet, and asked their opinion, via multiple-choice survey questions, regarding when and how the intervention should be delivered.

The secondary outcome was the proportion of participants who agreed or strongly agreed that the intervention was upsetting. Participants who found the intervention upsetting were asked to discuss what they found upsetting via an open-ended question. Transcribed narrative responses to this question were analyzed using a limited application of the framework method[40] with A.E.T., C.M.C., and R.K.C each independently applying open coding, then agreeing on themes, and finally independently charting the themes present in each response.

Participants who agreed or strongly agreed that the intervention was upsetting versus those who did not agree were compared for differences using descriptive statistics, the Wilcoxon-Mann-Whitney two-sample test for continuous variables, the Chi-square test for categorical values, and the Fisher’s exact test when cells counts were <10. The study was not powered or designed to test hypotheses about differences in the distribution of participant or patient characteristics; therefore, to encourage appropriate interpretation of statistical comparisons, we report both the p-value and the absolute value of the effect size (Cohen’s d) to provide an indication of the magnitude of effect.[41,42] The “Neither Agree or Disagree” response was combined with the Strongly agree and Agree responses as a sensitivity analysis. All statistics were generated using the R programming language version 3.3.2 (Vienna, Austria).

## Results

**Fig 1:**
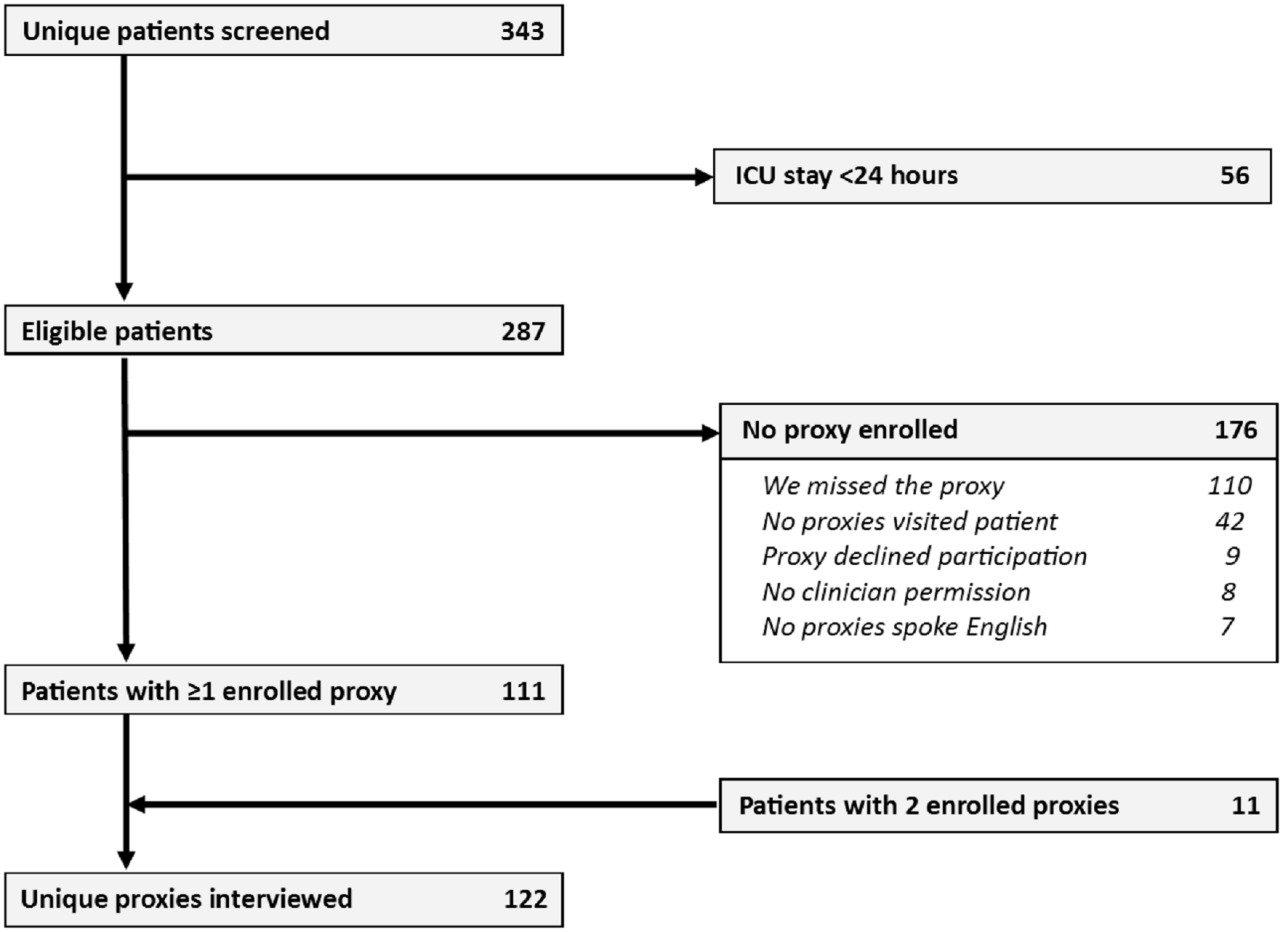
Study flow diagram.

We enrolled 122 proxies representing 111 eligible patients (Fig 1). Enrolled proxies had a median age of 51 years old (interquartile range (IQR) 39, 61) and 14 years of formal education (IQR 12, 16), with 83 (68%) female, 55 (45%) Black or African-American, and 79 (65%) the legal healthcare proxy according to Maryland law (Table 1). Patients had a median length of stay of 5 days (IQR 3,9) in the ICU and of 11 days (IQR 7, 25) in the hospital,(Table 2) with 101 patients (91%) living at home prior to hospitalization. ICU mortality was 18% and 37 patients (33%) died in hospital or were discharged to hospice.

**Table 1:** Characteristics of interviewed proxies.

Characteristic	N = 122
Age^a^, median (IQR)	51 (39, 61)
Female^a^, n (%)	83 (68%)
Self-identified race^a^, n (%)	
Black or African American	55 (45%)
White	55 (45%)
Other	8 (7%)
Years of education, median (IQR)	14 (12, 16)
Have you ever supported a loved one in an ICU before?^a^ Yes (%)	75 (61%)
Patient's legal surrogate decision-maker	79 (65%)
Relation to Patient^b^	
Daughter	31 (25%)
Female Spouse or Partner	31 (25%)
Male Spouse or Partner	15 (12%)
Parent	14 (11%)
Sibling	12 (10%)
Non-nuclear family member	10 (8%)
Son	9 (7%)

^**a**^ Proxies declined to report age (n = 2), sex (n = 2), race (n = 4), and previous experience as an ICU proxy (n = 3)

^**b**^ Percentages do not sum to 100% due to rounding

**Table 2:** Patient characteristics and outcomes.

Characteristic	N = 111
**Age**, Median (IQR)	58 (48,69)
**Female**, N (%)	56 (50%)
**Race**, N (%)	
White	49 (44%)
Black or African American	48 (43%)
Other	14 (13%)
**Median income of zip code**, $1000s of USD^a^, Median (IQR)	59.1 (36.0, 81.8)
**Location prior to hospitalization**^b^, N (%)	
House/Apt (independent)	72 (65%)
House/Apt (with assistance)	29 (26%)
Long-Term ventilator/acute rehabilitation/nursing home	9 (8%)
**Admission diagnosis**, N (%)	
Respiratory failure	49 (44%)
Sepsis	23 (21%)
Gastrointestinal	11 (10%)
Cardiovascular	6 (6%)
Other	22 (20%)
**Code Status after 24 hours in ICU**, N (%)	
Full code^c^	97 (87%)
Full code with specific treatment limitations (e.g., No hemodialysis)	6 (5%)
Do Not Resuscitate and Do Not Intubate	8 (7%)
**ICU length of stay in days**, median (IQR)	5 (3,9)
**Subsequent withdrawal of life-support in the ICU**^b^, N (%)	21 (19%)
**Subsequent death in ICU**, N (%)	20 (18%)
**Hospital length of stay in days**, median (IQR)	11 (7,25)
**Subsequent hospital discharge disposition**, N (%)	
House/Apt (independent)	34 (31%)
House/Apt (with home care)	14 (13%)
Died	29 (26%)
Hospice	8 (7%)
Other, including inpatient facilities	26 (23%)

**Abbreviation**: DNI, Do not intubate; DNR, Do not resuscitate; ICU, Intensive care unit; IQR, Interquartile Range

^**a**^ U.S. Census Bureau 2010–2014; $41,819 median household income for Baltimore City; $74,194 median household income for MD state. No zip code was provided for 1 patient from Saudi Arabia.

^**b**^ Missing for one patient

^**c**^ 51 (53%) of patients herein designated as full code didn’t have a documented code status 24 hours after ICU admission.

**Fig 2:**
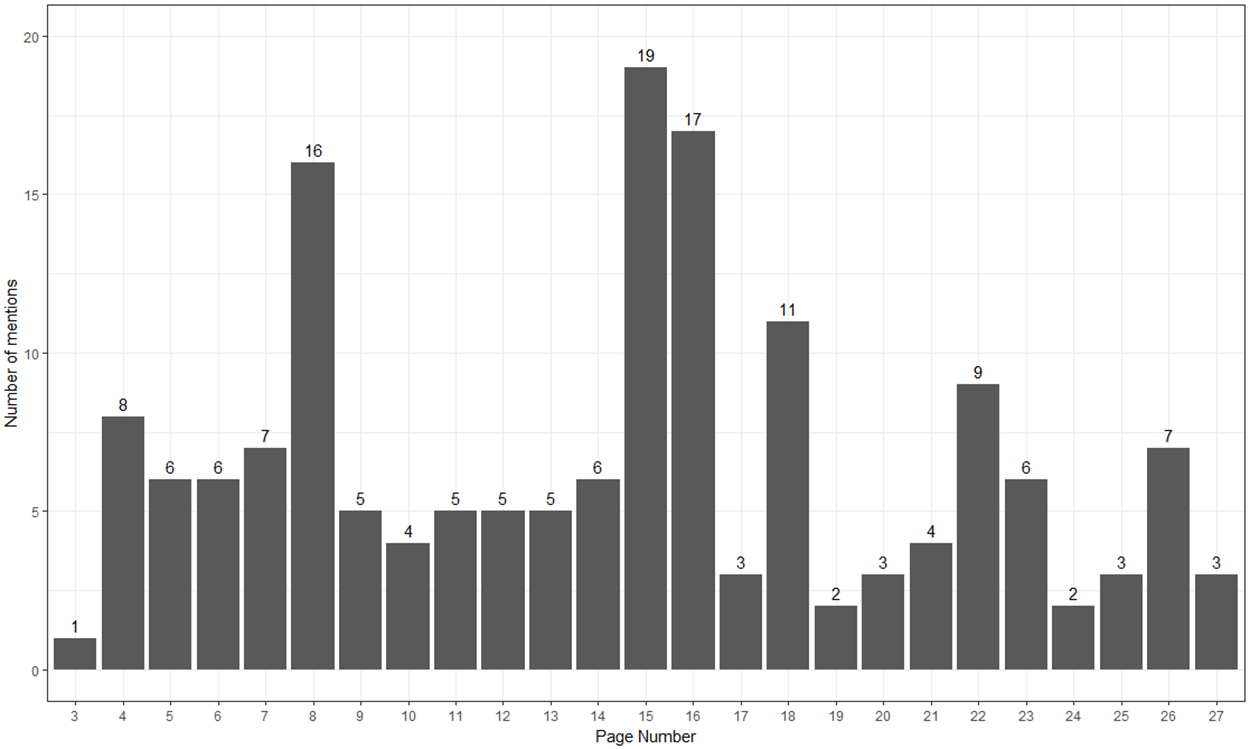
Important booklet pages according to ICU proxies.

Among the 122 proxies interviewed, 120 (98%, 95% CI 94% - 100%) agreed or strongly agreed that the intervention booklet was appropriate for adult friends and family of ICU patients, and 119 (98%, 95% CI 92% - 99%) agreed or strongly agreed that it is important for families to know the information in the booklet (Table 3). Although every page was cited by at least one proxy as being the most important, the most frequently cited pages page 15, which guides proxies who believe their loved one may be dying on what to ask their doctor, and page 16 which stresses that proxies are not alone and are welcome to work with their family or religious leader (Fig 2). Rather than citing a specific page as being important, many proxies instead indicated a section or theme of the booklet. The section with questions that proxies should ask was cited by 33 unique proxies (Table 4).

**Table 3:** Proxy responses to questions about booklet content and delivery.

The 3 questions below were asked of all **122** proxies.	No. proxies
*"The booklet is* ***appropriate*** *for adult friends and family-members of ICU patients*.*"* n (%)	
Strongly agree	46 (38%)
Agree	74 (61%)
Neither agree or disagree	0 (0%)
Disagree	2 (2%)
Strongly disagree	0 (0%)
*"It is* ***important*** *for families of ICU patients to know the information in the booklet*.*"* n (%)	
Strongly agree	69 (57%)
Agree	50 (41%)
Neither agree or disagree	2 (2%)
Disagree	1 (1%)
Strongly disagree	0 (0%)
*"Parts of the booklet are* ***upsetting***.*" n (%)*	
Strongly Agree	6 (5%)
Agree	48 (39%)
Neither agree or disagree	7 (6%)
Disagree	49 (40%)
Strongly Disagree	12 (10%)
The following question was asked of all proxies who agreed or strongly agreed that it is important for families of ICU patients to know the information in the booklet.	
*"In your opinion*, *which pages in the booklet are most important for ICU proxies to know*?*"* (list **all** pages mentioned)^a^	Number of mentions
Pages with background information about proxy decision-makers	42 (34%)
Pages that address formulating treatment goals	39 (32%)
Tips for being a great proxy and recommended questions to ask doctors	79 (65%)
Contact information for support resources (social work, chaplaincy, etc.)	21 (17%)
The 2 questions below were added to the interview in week 3 of the study and were only asked of proxies who agreed or strongly agreed that it was important for families to know the information in the booklet, generating **97** responses.	
*"In your opinion*, *when should ICU proxies receive the information in the booklet*?*"* n (%)	
As soon as they arrive in the ICU	57 (59%)
After they’ve had a chance to speak to a doctor about their loved ones care	33 (34%)
Only if their loved one’s health isn’t improving	6 (6%)
Declined	1 (1%)
*"In your opinion*, *what is the best way to share the information in the booklet with ICU proxies*?*"* n (%)	
In a face-to-face conversation	54 (56%)
On paper (for example the booklet)	32 (33%)
In a video (for example on an iPad or on YouTube)	9 (9%)
In a conversation on the phone	2 (2%)

^**a**^ While some proxies cited specific page numbers, others cited entire sections of the booklet such as "The goals section" or "The pages with questions to ask the doctors." The number of times individual pages in the booklet were cited is provided in Supplemental information.

**Table 4:** Proxies responses to the question: *“Which page or part of the booklet did you find upsetting and why was it upsetting*?*”* (N = 122).

Theme and exemplar quotes	Number of quotes
**Acknowledgement that critically ill patients may die**	15 (12%)
* “Anytime you talk about death that's always upsetting because people don't want to address that option*, *even though it is inevitable*.*”*	
* “The part that talked about the patient could die*.*”*	
**Page 15 which states**: “Some patients are so sick, that their goals cannot be reached. If you are worried that your loved one is dying and treatment will not help, ask a doctor: “Is it time to let go?”)	12 (10%)
* “Page 15—it’s hard to see*!*”*	
* “Page 15*. *It’s hard to ask the doctor*: *‘Is it time to give up*?*’”*	
**The section of the booklet containing examples of patient goals**	10 (8%)
* “The goals are sad*.*”*	
* “Reading about goals*.*”*	
**Making decisions**	9 (7%)
* “Just the fact that they* [proxies] *will have to make decisions*.*”*	
* “The reality of letting go and needing to make a decision*.*”*	
**Uncertainty about the patient’s prognosis**	8 (7%)
* “When it says [that] no one knows exactly what will happen*.*”*	
* “You never really know what will happen to someone you love*.*”*	
**Parts of the booklet related directly to the proxy’s loved one in the ICU**	7 (6%)
* “Page 23 because that is what it looked like when we were talking about whether to take him off the ventilator*.*”*	
* “Because we are here with a son who may die*.*”*	
**Acknowledgement that some patients aren’t able to return to living in their homes**	6 (5%)
* “Knowing that a patient’s not going to come home*. *I don’t like to hear that part*.*”*	
* “Thinking about if they won’t come home*.*”*	
**The situation faced by families in the ICU is upsetting–not the booklet content**	6 (5%)
* “The whole situation is upsetting*, *not one particular page*.*”*	
* “Some people don’t realize the seriousness of this situation*. *People could* *die*!*”*	
**Page 11 which states**: “But many people have problems that cannot be cured like severe lung disease, kidney failure, and end-stage cancer. These people have important goals too.”	6 (5%)
* “Page 11*, *because some of these run in my family*.*”*	
* “Page 11*. *My husband has some of those*.*”*	
**Use of the word “die”**	3 (2%)
* “The use of the word ‘die*.*’ It feels a little cold*. *Maybe use something with less of a punch like ‘passing*.*’”*	
* “All the pages that have the death word because it’s just too upsetting to think your loved one is going to die*.*”*	
**Negativity**	1 (1%)
* “Because it was negative*. *Because God can heal anything*. *It was negativity that you’re putting on people*.*”*	

Histogram of booklet pages cited by proxies in response to the question: “In your opinion, which pages in the booklet are most important for ICU proxies to know?” Many proxies cited multiple pages of the booklet as being important. The written content of each page is provided within Table B of S1 File. Complete content including images are available for download at www.piperscience.org/proxy-activation.

Of these same 122 proxies, 54 (44%, 95% CI 35%– 54%) agreed or strongly agreed that parts of the booklet are upsetting. Among the proxies who found the intervention upsetting, 51 (94%) agreed or strongly agreed that the intervention was simultaneously acceptable and important. In fact, 5 of the 6 proxies who strongly agreed that the booklet was upsetting, also strongly agreed that it is important for families to know the information in the booklet. Among proxies asked to elaborate on what was upsetting, the most common theme mentioned by 15 proxies (12%, 95% CI 7%– 20%) was acknowledgment that critically ill patients may die, and the second most common theme cited by 12 proxies (10%, 95% CI 5% - 17%) related to page 15 (Table 4).

When compared to the 68 proxies who did not find the booklet upsetting, proxies who found the booklet upsetting were not statistically or substantially different in age, sex, education level, race, relation to the patient, ICU day at time of the interview, perceived decision-making authority, or prior experience in ICUs (absolute value of all effect sizes ≤0.38) (Table 5). The patients whose proxies who found the booklet upsetting were of similar age, lived in zip codes with similar median incomes, and experienced similar rates of hospital mortality (absolute value of all effect sizes ≤0.32). Results were qualitatively similar in the sensitivity analysis (Table C of S1 File).

**Table 5:** Participant characteristics by response to the statement: "Parts of the booklet are upsetting".

	Strongly Agree or Agree	Strongly Disagree, Disagree, Neither	P-value^a^	Absolute effect size^a^
**Proxy and interview characteristics**	**(N = 54)**	**(N = 68)**		
Age, median (IQR)^b^	47 (34, 60)	54 (46, 63)	0.04	0.38
Female, n (%)^b^	37 (70%)	46 (69%)	1.00	0.03
Years of education, median (IQR)	14 (12, 16)	14 (12, 16)	0.20	0.10
Self-identified race, n (%)^b^				
Black or African American	22 (41%)	33 (49%)	0.50	0.29
White	26 (48%)	29 (43%)		
Other	2 (4%)	6 (9%)		
Relation to Patient, n (%)^c^				
Spouse/Partner	21 (39%)	25 (37%)	0.55	0.06
Adult child	17 (32%)	23 (34%)		
Parent	6 (11%)	8 (12%)		
Other	10 (19%)	12 (18%)		
ICU day at time of interview, median (IQR)	2 (1, 3)	2 (1, 3)	0.79	0.04
*"Do you have legal authority to speak for [patient's name] if he/she is unable to make decisions about medical treatment*?*"* This question was asked **BEFORE** the proxy viewed the booklet.				
Yes	33 (61%)	48 (71%)	0.36	0.21
*"Have you ever supported a loved one in an ICU before*?*"*, n (%)^b^				
Yes	33 (61%)	42 (62%)	0.89	0.06
**Patients characteristics & Outcomes**^d^				
Age, median (IQR)	58 (45, 69)	58 (50, 70)	0.41	0.14
Female, n (%)	25 (46%)	35 (51%)	0.70	0.10
Income of zip code in $1000s of USD, median (IQR)^e^	58.6 (36.0, 74.1)	59.1 (36.0, 82.0)	0.90	0.03
Is the patient "full code" during the interview?^f^				
Yes	43 (80%)	60 (88%)	0.29	0.32
Location prior to hospitalization, n (%)^b^				
House/Apartment (independent)	39 (72%)	42 (62%)	0.32	0.32
House/Apartment (with assistance)	13 (24%)	18 (26%)		
Other	2 (4%)	7 (10%)		
Admission diagnosis, n (%)				
Respiratory failure	27 (50%)	29 (43%)	0.28	0.35
Sepsis	10 (19%)	14 (21%)		
Gastrointestinal	7 (13%)	4 (6%)		
Other	10 (19%)	21 (31%)		
In-hospital death, n (%)	15 (28%)	19 (28%)	1.00	0.00

**Abbreviation**: ICU, Intensive care unit; IQR, Interquartile Range; USD, United States Dollar

^**a**^ Absolute effect size = absolute value of difference in means or proportions divided by standard error. P-values obtained from the Wilcoxon-Mann-Whitney two-sample test for continuous values, and the Chi-square test for categorical values with Fisher’s exact test for cell-sizes <10.

^**b**^ Proxies declined to report age (n = 2), sex (n = 2), race (n = 4), and prior experience as an ICU proxy (n = 3). Location prior to hospitalization missing for 1 patient.

^**c**^ Percentages do not sum to 100% due to rounding.

^**d**^ 2 proxies were interviewed for 11 patients, creating 11 pairs of proxies independently answering questions about the same patient at different times during the ICU stay. In 4 dyads, the proxies gave discordant responses about whether the booklet was upsetting.

^**e**^ US Census Bureau 2010–2014; $41,819 median household income for Baltimore City; $74,194 median household income for MD state. No zip code was provided for 1 international patient.

^**f**^ 54 (53%) of patients who are herein designated as full code didn’t have any documented code status during the interview.

There were 97 proxies enrolled after the third week of the study who agreed or strongly agreed that the study information was important. Asked when and how the information in the intervention should be conveyed, 57 (59%, 95% CI 48% - 69%) said as soon as the proxy arrived in the ICU, 33 (34%, 95% CI 25% - 44%) said after the proxy spoke to a doctor about their loved ones care, and 6 (6%, 95% CI 3% - 14%) said only if their loved ones health was not improving. When asked about how to provide the information, 54 (56%, 95% CI 45% - 66%) wanted to have a face-to-face conversation, 32 (33%, 95% CI 24% - 43%) wanted to receive a paper version of the booklet and 9 (9%, 95% CI 5% - 17%) preferred viewing a video.

## Discussion

In this Phase I study of an intervention to educate ICU families about the role of a healthcare proxy and prepare them to communicate effectively with clinicians, 98% of proxies rated the intervention as both appropriate and important, and 44% also found the intervention upsetting. Proxies considered recommended questions for intensivists the most important information within the intervention. Demographics of proxies and patients were similar comparing proxies who did versus did not find the intervention upsetting.

Providing ICU families with an informational leaflet is endorsed by the Agency for Healthcare Research and Quality as a process measure of high quality palliative care in the ICU [43]. Previous leaflets designed for use in the ICU [29,44–47] have largely targeted the families of dying patients, and provided information about the clinical environment such as commonly used medical jargon, procedures, ICU rules, and explanations of the equipment in a patient’s room. Our intervention was markedly different from previous leaflets in four important ways. First, our intervention was designed to be appropriate for all adult family members of ICU patients regardless of survival prognosis. Second, the booklet in our intervention functioned not as information that a family could choose to access, but as a script that was read aloud and verbatim to each participant. Reading aloud demonstrates that saying the words “death” and “dying” is acceptable in the ICU setting and ensures that all participants receive the same intervention regardless of available time, interest, or health literacy. Third, the intervention directly addressed that not all ICU patients survive or recover sufficiently to live at home, thereby helping to set appropriate expectations. Finally, our intervention made no attempt to explain a patient’s diagnosis, clinical care, prognosis, or treatment options; rather, we focused on **how** proxies should attempt to obtain this information from ICU clinicians.

Providing families with information is not sufficient to ensure engagement or shared decision-making [48]. Those who choose to be proxies must see themselves as active participants, bestowed with the authority to convey information about the patient’s goals and values, as well as a responsibility to obtain information about prognosis and treatment options. This challenge requires redefining the family member’s role, similar to patient activation in the outpatient setting [49,50]. We addressed this challenge by describing ICU proxies as people who volunteer for a responsibility that is respected, psychologically taxing, and vitally important.

The high prevalence of proxies rating the intervention as upsetting and the inability to anticipate which proxies will be upset means that the intervention booklet should not be left in waiting rooms. Although 56% of proxies said they wanted to receive information via a face-to-face conversation, we believe this rate would have been even higher if proxies had been permitted to select multiple responses to this question since many proxies said they preferred to have both a face-to-face conversation and receive written material that could be shared with other family members. As one proxy explained: *“It’s really important to talk to someone*. *I would have thrown this away if it had just been handed to me on admission*.*”*

This Phase I study had potential limitations. It was conducted in a single ICU and excluded families who did not physically visit the ICU or did not speak English. Similar interventions will need to be tailored to their jurisdiction given that statutes about who can serve as a proxy for patients who lack decisional capacity vary widely across states.[51] The study also only addressed proxy’s immediate reactions to the intervention which may have changed over time, particularly after discharge. Further study is needed to determine whether describing parts of the booklet as “upsetting” immediately following the intervention correlates with any long-term mental health symptoms.[52,53] As a Phase I study there was no attempt to estimate the intervention’s impact on proxy behavior or patient care. Despite these limitations, the study has important strengths including being designed to ensure all participants received the same exposure to the intervention, being conducted in a socio-economically diverse population, and being developed with input from ICU proxies and a large multi-disciplinary group of relevant stakeholders.

Many questions remain to be answered about this intervention. The most pressing question is whether it impacts proxy behavior and communication. Additional research is needed to determine if the intervention improves proxy activation. There are also logistical questions, namely who should deliver the intervention, as well as issues of cost-effectiveness. If the intervention positively impacts proxy behavior and is cost-neutral, it will face the same challenges which limit the use of interventions designed to foster patient/family engagement and shared decision-making, namely, resistance to change and the lack of reward for clinical utilization [54].

## Conclusions

This Phase I, single-center study of a brief intervention designed to set expectations, explain an ICU proxy’s role in the ICU setting, and prepare proxies to communicate effectively with clinicians was acceptable and important to nearly all 122 participating ICU proxies. Frequently, the intervention was simultaneously rated as upsetting by these same proxies, with neither patient nor proxy characteristics associated with this finding. This intervention represents the early stages of an attempt to adapt the principles of patient activation to the ICU setting. Additional research is required to determine if this approach impacts proxy behavior.

## Supporting information

S1 File Supporting information. (PDF)
