# Supplementary material for: A brief intervention for preparing ICU families to be proxies: A phase I study
Source: PLoS One. 2017 Oct 2;12(10):e0185483. doi: 10.1371/journal.pone.0185483 (PMC5624606; doi:10.1371/journal.pone.0185483)
Supplement: S1 File — (PDF) [file pone.0185483.s001.pdf]

Table A: Additional methodological details

**Recruitment**	Study team members approached eligible families daily until 1)the family declined participation, 2)the patient was discharged from the MICU, 3)the patient died, or 4)7 days had passed since the family became eligible. Families who had not participated could regain eligibility, if a discharged patient was re-admitted to the MICU. Family members <18 year old, those that did not understand or speak English, and those not physically present in the MICU were not eligible. If a legal proxy did not wish to participate or was not expected to visit the ICU, an adult family member identified as participating in surrogate decision-making by a member of the MICU clinical team was approached for enrollment.
**Data collection**	The following patient characteristics and outcomes were collected via chart review: patient age, sex, race, median income of home zip code, residential location prior to hospitalization (house or apartment independently, house or apartment with assistance, residential care), ICU admission diagnosis, code status after 24 hours in the MICU, MICU length of stay, MICU mortality, hospital mortality, and hospital discharge disposition. Participating family members were asked to report their age, sex, race, years of formal education, relationship to the patient, and previous experience supporting a loved one in an ICU.

Table B: Thematic responses to the question: *"In your opinion, which pages in the booklet are most important for ICU proxies to know?"*

Page Content	Section or theme	No. of mentions
**Page 3:** Someone you care about is in the Intensive Care Unit or I.C.U. The ICU is a special part of the hospital. Some people in the ICU are sick enough to die.		
**Page 4:** Many ICU patients are too sick to talk. If your loved one didn't choose someone to speak for them, a Maryland law says who may speak for them.	"The part about 'You don't have to be The Proxy' "	10
**Page 5:** The person who speaks for the patient is called: **THE PROXY**		
**Page 6: WAIT! What if I don't want to be The Proxy?!**		
**Page 7: You don't have to be The Proxy.** If you don't want to be The Proxy, you should tell a doctor, nurse, or social worker. They will help you find someone else to be The Proxy.		
**Page 8:** If you are The Proxy, you have a **BIG** job. **The Proxy's job is to tell doctors about the sick person's goals.** Doctors need this information. It's ok if you're not sure what your loved one would say. It's also ok for goals to change over time.	"The part about the goals."	21
**Page 9:** [picture with icon of proxy saying] "I think his goal is to…"		
**Page 10:** For some people, the goal is to be 100% cured.		
**Page 11:** But many people have problems that cannot be cured like severe lung disease, kidney failure, and end-stage cancer. These people have important goals too.		
**Page 12:** For example, sometimes the goal is to return to a job or hobby the patient loves.		
**Page 13:** For some people, the goal is to live long enough to see an important event, like a graduation, a birth, a wedding, or the Ravens post-season.		
**Page 14:** For other people, the goal is to be awake and comfortable before they die.		
**Page 15:** Some patients are so sick, that their goals cannot be reached. If you are worried that your loved one is dying and treatment will not help, ask a doctor: **"Is it time to let go?"**		
**Page 16:** Proxies are not alone. Most Proxies work with their family. Some Proxies work with their priest, pastor, rabbi, imam, or faith leader. All religious leaders are welcome in the ICU. There are also chaplains in the hospital 24-hours a day.		
**Page 17:** Some Proxies want to look things up and make decisions on their own. That's OK.		
**Page 18:** Other Proxies want doctors to make recommendations. That's also OK. Ask: **"What do you recommend?"** or **"What would you do if she was your family?"**		
**Page 19:** Here are **6 tips** for being a great Proxy.	"The 6 tips for being a great Proxy"	22
**Page 20: #1 Explain your loved one's goals.** When you meet a new doctor or nurse, tell them you're The Proxy. Don't assume they know you, or what matters to your loved one.		
**Page 21: #2 Give doctors phone number to call** or explain the best way to reach you. It is VERY important they know how to reach you in an emergency.		
**Page 22: #3 Ask doctors: "What's most likely to happen?"** Nobody know exactly what will happen. But you need to know if your loved one may recover, never return home, or die.		
**Page 23: #4** You may be asked if your loved one would want specific treatments or tests. This can be a hard question. **Ask: 1."Will it help her reach her goal?" 2."What are her other options?"**		
**Page 24: #5 Meet the Social Worker in the ICU.** She can help you and your loved one get the support you need.		
**Page 25: #6 Make sure you sleep and eat to stay healthy.** Being The Proxy can be very stressful.		
**Page 26: 4 questions ICU proxies should ask 1. What is most likely to happen? 2. Will treatment help reach his goal? 3. What are her other options? 4. What do you recommend?**	"The 4 questions"	33
**Page 27: Contact information | Social Work** Amy (xxx) xxx-xxxx, Jeanne-Marie (xxx) xxx-xxxx, 9am – 5pm, Mon – Fri **| Hospital Chaplains** Ask any nurse in the ICU 24 hours **| Patient Relations** (xxx) xxx-xxxx 9am – 5pm, Mon – Fri **| Johns Hopkins Patient-Family Advisory Council** patientfamilycouncil@jhmi.edu	"The phone numbers"	15

Table C: Sensitivity analysis of participant characteristics by response to the statement: "Parts of the Booklet are Upsetting"

	Strongly Agree or Agree, Neither	Strongly Disagree, Disagree	P-value[a]	Absolute effect size[a]
**Proxy and interview characteristics**	**(N = 61)**	**(N = 61)**		
Age, median (IQR)[b]	47 (34, 60)	54 (46,63)	0.02	0.43
Female, n (%)[b]	42 (69%)	41 (67%)	0.78	0.09
Years of education, median (IQR)	14 (12, 16)	14 (12, 16)	0.18	0.15
Self-identified race, n (%)[b]				
Black or African American	25 (41%)	30 (49%)		
White	29 (48%)	26 (43%)	0.60	0.17
Other	3 (5%)	5 (8%)		
Relation to Patient, n (%)[c]				
Spouse/Partner	22 (36%)	24 (39%)		
Adult child	22 (36%)	18 (30%)	0.88	0.15
Parent	7 (11%)	7 (11%)		
Other	10 (16%)	12 (20%)		
ICU day at time of interview, median (IQR)	2 (1, 3)	2 (1, 3)	0.79	0.04
"Do you have legal authority to speak for [patient's name] if he/she is unable to make decisions about medical treatment?" This question was asked **BEFORE** the proxy viewed the booklet.				
Yes	36 (59%)	45 (74%)	0.13	0.33
"Have you ever supported a loved one in an ICU before?", n (%)[b]				
Yes	38 (62%)	37 (61%)	0.72	0.10
**Patients characteristics & Outcomes[d]**				
Age, median (IQR)	58 (48, 69)	58 (48, 70)	0.73	0.06
Female, n (%)	29 (48%)	31 (51%)	0.86	0.07
Income of zip code in $1000s of USD, median (IQR)[e]	58.6 (35.7, 72.0)	59.9 (36.0, 86.1)	0.53	0.07
Is the patient "full code" during the interview?[f]				
Yes	48 (79%)	55 (90%)	0.13	0.44
Location prior to hospitalization, n (%)[b]				
House/Apartment (independent)	42 (69%)	39 (64%)		
House/Apartment (with assistance)	15 (25%)	16 (26%)	0.87	0.32
Other	4 (7%)	5 (8%)		
Admission diagnosis, n (%)				
Respiratory failure	29 (48%)	27 (44%)		
Sepsis	14 (23%)	10 (16%)	0.24	0.35
Gastrointestinal	7 (11%)	4 (7%)		
Other	11 (18%)	20 (33%)		
In-hospital death, n (%)	17 (28%)	17 (28%)	1.00	0.00

Abbreviation: ICU, Intensive care unit; IQR, Interquartile Range; USD, United States Dollar

a Absolute effect size = absolute value of difference in means or proportions divided by standard error. P-values obtained from the Wilcoxon-Mann-Whitney two-sample test for continuous values, and the Chi-square test for categorical values with Fisher's exact test for cell-sizes <10.

b Proxies declined to report age (n = 2), sex (n = 2), race (n = 4), and prior experience as an ICU proxy (n=3). Location prior to hospitalization missing for 1 patient.

c Percentages do not sum to 100% due to rounding.

d 2 proxies were interviewed for 11 patients, creating 11 pairs of proxies independently answering questions about the same patient at different times during the ICU stay. In 4 dyads, the proxies gave discordant responses about whether the booklet was upsetting.

e US Census Bureau 2010-2014; $41,819 median household income for Baltimore City; $74,194 median household income for MD state. No zip code was provided for 1 international patient.

f 54 (53%) of patients who are herein designated as full code didn't have any documented code status during the interview.
